# Theoretical study of charge-transport and optical properties of organic crystals: 4,5,9,10-pyrenedi­imides

**DOI:** 10.1107/S2052252519004706

**Published:** 2019-05-09

**Authors:** Jin-Dou Huang, Kun Yu, Xiaohua Huang, Dengyi Chen, Jing Wen, Shibo Cheng, Huipeng Ma

**Affiliations:** a Key Laboratory of New Energy and Rare Earth Resource Utilization of State Ethnic Affairs Commission, School of Physics and Materials Engineering, Dalian Nationalities University, Dalian 116600, People’s Republic of China; b College of Medical Laboratory Science, Dalian Medical University, Dalian 116044, People’s Republic of China; c School of Chemistry and Chemical Engineering, Shandong University, Jinan 250100, People’s Republic of China

**Keywords:** pyrenedi­imides, charge-carrier mobility, time-dependent density functional theory, structure–property relationships

## Abstract

This work presents a systematic study of the conducting and optical properties of a family of aromatic di­imides reported recently and discusses the influences of side-chain substitution on the reorganization energies, crystal packing, electronic couplings and charge injection barrier of 4,5,9,10-pyrenedi­imide (PyDI). Quantum-chemical calculations combined with the Marcus–Hush electron transfer theory revealed that the introduction of a side chain into 4,5,9,10-pyrenedi­imide increases intermolecular steric interactions and hinders close intermolecular π–π stacking, which results in weak electronic couplings and finally causes lower intrinsic hole and electron mobility in *t*-C_5_-PyDI (μ_h_ = 0.004 cm^2^ V^−1^ s^−1^ and μ_e_ = 0.00003 cm^2^ V^−1^ s^−1^) than in the C_5_-PyDI crystal (μ_h_ = 0.16 cm^2^ V^−1^ s^−1^ and μ_e_ = 0.08 cm^2^ V^−1^ s^−1^). Furthermore, electronic spectra of C_5_-PyDI were simulated and time-dependent density functional theory calculation results showed that the predicted fluorescence maximum of *t*-C_5_-PyDI, corresponding to an *S*_1_→*S*_0_ transition process, is located at 485 nm, which is close to the experimental value (480 nm).

## Abstract 2

The shapes of the HOMOs/LUMOs and the relative positions of adjacent molecules decide the size of the intermolecular electronic couplings.

## 1. Introduction   

**Figure 1:**
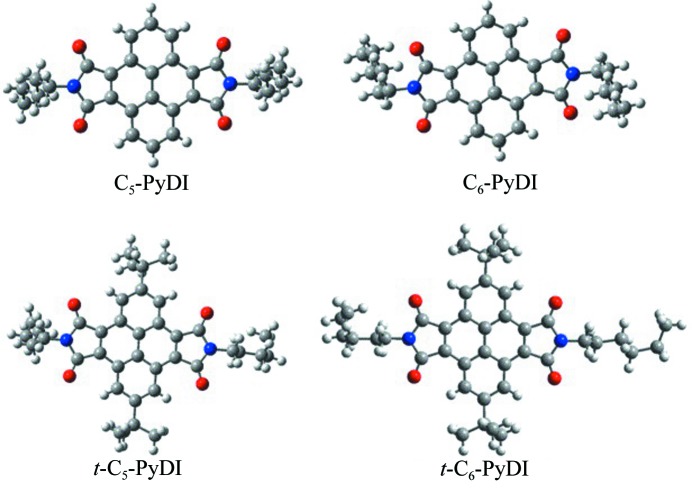
Molecular structures of C_5_-PyDI, C_6_-PyDI, *t*-C_5_-PyDI and *t*-C_6_-PyDI. Red: O; blue: N; gray: C; white: H.

Organic semiconductors are of great interest for their intrinsic scientific challenge and potential applications in organic electronic devices such as organic light-emitting diodes (Lin *et al.*, 2016 ▸), plastic solar cells (Gao *et al.*, 2017 ▸; Skrypnychuk *et al.*, 2016 ▸; Yang *et al.*, 2016 ▸), organic lasers (Kuehne & Gather, 2016 ▸; Zhang *et al.*, 2016 ▸), (bio)chemical sensors (Wang *et al.*, 2017 ▸; Haughey *et al.*, 2016 ▸) and organic field-effect transistors (OFETs) (Zhao *et al.*, 2017 ▸; Sung *et al.*, 2016 ▸; Raghuwanshi *et al.*, 2016 ▸; Matsushima *et al.*, 2016 ▸; Ford *et al.*, 2016 ▸). Compared with *p*-channel materials, the development of high-performance ambient-stable *n*-channel materials has largely lagged due to the fact that the transport in *n*-channel conductors is degraded easily by air, and their low electronic affinity hinders efficient injection of electrons into the empty lowest unoccupied molecular orbital (LUMO). In recent years, numerous attempts were made to overcome these difficulties, and some new *n*-channel semiconductors have been realized via functionalization of Naphthalene di­imides (NDIs) (Bélanger-Chabot *et al.*, 2017 ▸; Yuan *et al.*, 2016 ▸; Purdum *et al.*, 2016 ▸; Kobaisi *et al.*, 2016 ▸), diketo­pyrrolo­pyrrole (DPP) (Tang *et al.*, 2017 ▸; Yao *et al.*, 2016 ▸; Li *et al.*, 2016 ▸; Yi *et al.*, 2015 ▸), perylene di­imides (Yue *et al.*, 2014 ▸; Liu *et al.*, 2014 ▸; Zhang & Zhao, 2012 ▸; Wuerthner & Stolte, 2011 ▸) and heteroacenes (Xu *et al.*, 2016 ▸) with electron-withdrawing substituents or alkyl chains. For example, Yuan *et al.* synthesized di­fluoro- and tetra­fluoro-substituted Naphthalene di­imides, and the OFETs based on these fluorinated NDIs exhibited *n*-channel field-effect character under ambient conditions with a maximum mobility of 0.1 cm^2^ V^−1^ s^−1^ (Yuan *et al.*, 2016 ▸). Klauk and co-workers found that halogen and cyano substituents are ideally suited to tune the DPP dyes’ absorption and redox properties into a favorable regime for applications in organic transistors and solar cells, and the alkyl chain substitution could lead to superior π–π contacts between DPP molecules within the layers and closer distances between the DPP layers, both of which are favorable for charge-carrier transport (Stolte *et al.*, 2016 ▸). In our previous work, the transport parameters of the cyanated bi­thio­phene-functionalized DPP molecule were simulated in the context of the band model and hopping models, and the theoretical intrinsic electron mobility could reach 2.26 cm^2^ V^−1^ s^−1^ (Huang *et al.*, 2015 ▸). More recently, Zhang *et al.* designed and synthesized a new type of aromatic di­imide, pyrene-di­imide (PyDI) small molecules (see Fig. 1 ▸), which exhibit excellent charge-transport capability and attractive optical properties (Wu *et al.*, 2017 ▸). As a potential family of *n*-type organic semiconductors however, there are very few reports on their structure–function relationship and electronic structural properties up to now. A theoretical investigation of the relationship between the nature of the different alkyl groups and device performance, and a prediction of their intrinsic electron-transfer mobility are instrumental in guiding the molecular design and development of novel organic semiconducting materials, which is also the focus of this work.

In this work, the charge-transport and optical properties of 4,5,9,10-pyrenedi­imides (C_5_-PyDI, C_6_-PyDI, *t*-C_5_-PyDI and *t*-C_6_-PyDI, see Fig. 1 ▸) were systematically investigated. Firstly, the reorganization energy associated with charge transport was evaluated using the adiabatic potential energy surface (APS) method, and the influence of the bond-parameter variations on the local electron–vibration coupling were discussed in detail, which evaluates well the effects of different alkyl groups on the reorganization energy. Then, the structural and electronic properties of C_5_-PyDI, C_6_-PyDI, *t*-C_5_-PyDI and *t*-C_6_-PyDI were investigated, and the angular resolution anisotropic mobility for both electron and hole transport were further evaluated using the newly developed simulation methods. In the end, the steady-state absorption and fluorescence spectra were simulated and a new assignment of fluorescence bands in the experiments is confirmed.

## 2. Computational methods   

### 2.1. Organization energy, ionization potential and electronic affinity   

The reorganization energy λ, associated with the charge-transport process in organic solid materials, can be evaluated in two ways (Zhao & Liang, 2012 ▸). The first is the normal-mode (NM) analysis method, which partitions the total relaxation energy into contributions from each vibrational mode:
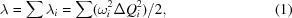
where Δ*Q*_*i*_ represents the displacement along normal mode *Q*_*i*_ between the equilibrium geometries of the neutral and charged molecules; ω_*i*_ is the corresponding frequency. The other method is the APS method (the four-point approach), in which λ can be expressed as follows:
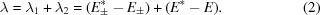
Here, *E* and *E*_±_ represent the energies of the neutral and cation/anion species in their lowest energy geometries, respectively; *E* ^*^ and 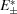 are the energies of the neutral and cation/anion species with the geometries of the cation/anion and neutral species, respectively. Our previous studies showed that the APS method is suitable for both flexible and rigid molecules; in comparison, the NM analysis is more appropriate for rigid molecules due to the large deviation of the lattice vibration from the harmonic oscillator model for flexible molecules (Ma *et al.*, 2017*a* ▸,*b* ▸). In this work, we selected the APS method to calculate the reorganization energy.

From the APS of neutral/charged species, the vertical ionization potential (VIP), adiabatic ionization potential (AIP), vertical electronic affinity (VEA) and adiabatic electron affinity (AEA) can be calculated as
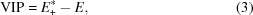


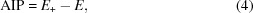


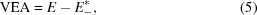


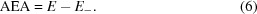
Full-geometry optimizations of the monomer molecules and the reorganization energy calculations are carried out using the B3LYP functional (Lee *et al.*, 1988 ▸) in conjunction with the 6-311G** basis set. These calculations are performed with the *GAUSSIAN09* package (Frisch *et al.*, 2009 ▸).

### 2.2. Electronic coupling   

The intermolecular electronic coupling *V*_*ij*_ from site *i* to site *j*, which describes the overlap of electronic wavefunctions between the donor and acceptor states, can be written as
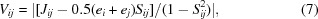
where *S*_*ij*_, *J*_*ij*_ and *e*_*i*(*j*)_ represent the spatial overlap, charge transfer integrals and site energies, respectively. These physical quantities can be calculated as follows:
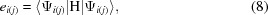


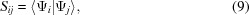


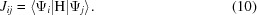
Here, H is the Kohn–Sham Hamiltonian of the dimer system and Ψ_*i*(*j*)_ represents the monomer HOMOs (for hole transport) or LUMOs (for electron transport) with Löwdin’s symmetric transformation, which can be used as the orthogonal basis set for calculation. The calculations of all electronic couplings in different molecular dimers are performed with the PW91/TZVP of density functional theory (DFT) implemented in the Amsterdam density functional (ADF) program (te Velde *et al.*, 2001 ▸).

### 2.3. Anisotropic mobility   

The anisotropic mobility is an important intrinsic property of charge transport in organic semiconductors, which depends significantly on the specific surface of organic crystals. Herein, we simulated the angle-resolved charge mobility of 4,5,9,10-pyrenedi­imides by means of solving the master equation, which has been described in detail elsewhere (Yin & Lv, 2008 ▸; Yin *et al.*, 2012 ▸). Charge-transfer (CT) kinetics through a solid material with many possible residence sites can be described by the master equation:
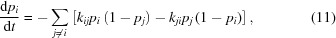
where *k*_*ij*_ is the CT rate constant from site *i* to site *j* in the crystal considering the correction of the electronic field, *p*_*i*_ is the charge occupied density on site *i*, and 1 − *p*_*i*_ is the Coulomb penalty factor, which prevents two or more charges at the same time from occupying the same site. If the CT reaches the so-called steady state, d*p*_*i*_/d*t* = 0, the *p*_*i*_ can be obtained by an efficient iterative procedure given a full set of CT constant *k*_*ij*_ values. When an external electronic field *E* is applied to the crystal, the charge will drift accordingly, and the charge mobility *m* can be determined from the velocity *v* as the linear response of the motion to the perturbation:
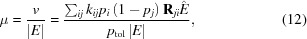
where 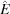 is the unit vector of the applied electric field, **R**_*ji*_ is the vector from site *i* to site *j* and *p*_tol_ is the total charge population in the investigated supercell. The calculation here is performed using the periodic boundary condition with a supercell of size 3 × 3 × 3, and the external electric field *E* is set to a relatively small value of 1.0 × 10^−3^ V Å^−1^.

### 2.4. Electronic spectra   

As the accuracy of a TDDFT calculation is strongly dependent on the chromophore family, it is essential that TDDFT results are validated by comparing the experimental data prior to a detailed interpretation. The accuracies of different functionals such as B3LYP, PBE0 and M06-2x were assessed by comparing the predicted wavelengths and intensities of the lowest energy bands with experimental absorption data. Our tests showed that the simulated absorption spectra based on the geometry structures optimized using M06-2x are in better agreement with the experimental UV–vis spectra, which is inconsistent with previous studies (Huang *et al.*, 2017a ▸,*b* ▸, 2018 ▸; Ma & Huang, 2016 ▸; Yang *et al.*, 2018 ▸, 2017 ▸). Therefore, the geometry optimizations of *t*-C_5_-PyDI at the *S*_0_ state and the *S*_1_ state were implemented using the DFT and TDDFT methods at the M06-2x/TZVP (triple-zeta valence quality with one set of polarization functions) level (Treutler & Ahlrichs, 1995 ▸). The self-consistent field convergence thresholds of the energy for both the ground-state and excited-state optimization were used as the default settings (10^−6^). The excited-state Hessian was obtained by numerical differentiation of analytical gradients using central differences and default displacements of 0.02 Bohr. The geometry optimizations were performed without constraints on bond lengths, angles or dihedral angles. All local minima were confirmed by the absence of an imaginary mode in the vibrational analysis calculations. To evaluate the solvent effect, cyclo­hexane and chloro­form were selected as the solvent in the calculations using the conductor-like screening model (COSMO) method (Klamt & Schüürmann, 1993 ▸).

## 3. Results and discussion   

### 3.1. Reorganization energy   

**Figure 2:**
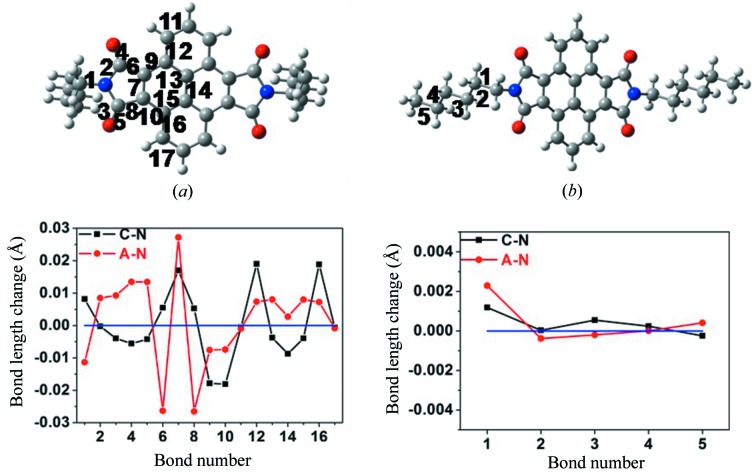
Calculated variations in the bond lengths of isolated C_6_-PyDI upon oxidation (black symbols) and reduction (red symbols), the *x* axis represents the chemical bond (C—C, C—O and C—N) number that is marked using different numbers.

As one of the key parameters influencing the intrinsic charge-transport rates, the reorganization energies evaluated from the four-point approach are collected in Table 1 ▸. Comparison of the reorganization energies of C_5_-PyDI, C_6_-PyDI, *t*-C_5_-PyDI and *t*-C_6_-PyDI shows that the variation of alkyl chain length has little influence on the reorganization energies associated with intermolecular electron-transfer (λ_e_) and intermolecular hole transfer (λ_h_), and the introduction of *t*-butyl groups reduces the λ_h_ and λ_e_ values no more than 0.01 eV. For the studied compounds, the λ_e_ values are much larger than the ones associated with hole-transfer (λ_h_). As show in Table 1 ▸, the λ_e_ values of C_5_-PyDI, C_6_-PyDI, *t*-C_5_-PyDI and *t*-C_6_-PyDI are 0.258, 0.257, 0.251 and 0.251 eV, respectively, which are about 0.07–0.09 eV larger than the corresponding λ_h_ values. This indicates that the pyrenedi­imide framework undergoes larger geometry relaxations during the electron-transfer process. For the sake of comprehensive analysis of the relationship between the molecular structure and the reorganization energy, we display the bond length alternation in the C_6_-PyDI molecule at oxidation and reduction, as shown in Fig. 2 ▸. For bonds 1 to 8, as shown in Fig. 2 ▸(*a*), the geometric relaxation occurs predominantly on electron transfer; in contrast, the smaller geometric changes in these bonds on hole transfer contribute less to λ_h_, which is consistent with the observation of a larger values of λ_e_ than λ_h_. For C—C bonds 9 to 17 in the pyrene core, the bond relaxation on hole transfer is more pronounced than those on electron transfer [see Fig. 2 ▸(*a*)], which means that the changes in these C—C bond lengths contribute more to λ_h_ than to λ_e_. Combined with the calculated λ_h_ and λ_e_ values, it can be concluded that the large λ_e_ value is mainly attributed to the geometric relaxations of five-membered imide rings and C=O bonds rather than the pyrene core. Moreover, the changes in C—C bond length in the alkyl chains are also depicted in Fig. 2 ▸(*b*). All bond-length variations of the C—C bonds between the neutral and charged forms are less than 0.003 Å, suggesting that the geometry relaxations of C—C bonds accompanied with hole and electron transport have a very small effect on their reorganization energies. This explains the small difference in the reorganization energies of C_5_-PyDI, C_6_-PyDI, *t*-C_5_-PyDI and *t*-C_6_-PyDI.

### 3.2. Crystal structure and electronic coupling   

**Figure 3:**
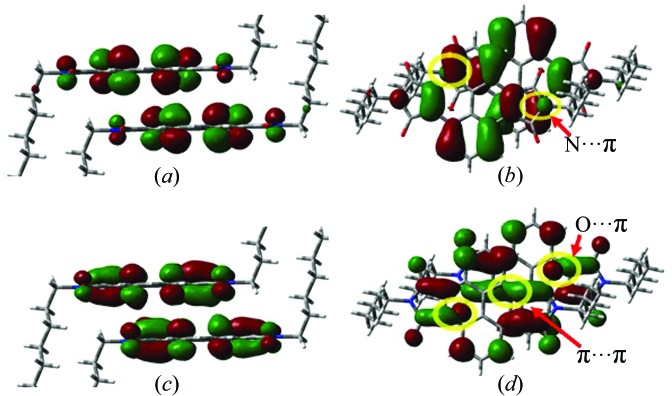
HOMOs (0.02 a.u.) for the P dimer of C_6_-PyDI in (*a*) side view and in (*b*) top view, and LUMOs (0.02 a.u.) for the P dimer of C_6_-PyDI in (*c*) side view and in (*d*) top view.

In the crystal structures of C_5_-PyDI and C_6_-PyDI, the molecules crystallize in a triclinic system and show a typical one-dimensional π-stacking motif, which is usually considered to exhibit strong π–π interactions and good charge-transport properties. In this motif, only face-to-face intermolecular packing modes can be formed. To facilitate the discussion below, this intermolecular packing mode is defined as the P dimer. For C_5_-PyDI and C_6_-PyDI, the calculated electronic couplings for hole and electron transfer (denoted as *V*_h_ and *V*_e_) and the mass-centered distances *r* in the P dimer are summarized in Table 2 ▸. It can be seen that intermolecular electronic couplings are relatively small even though the intermolecular distances are very short. For C_5_-PyDI, the *V*_h_ and *V*_e_ values are similar, both of which are less than 20 m eV; for C_6_-PyDI, the *V*_e_ value is 26.3 meV and *V*_h_ is only 7.2 meV. In order to understand the weak electronic couplings in the one-dimensional π-stacking motif, the shapes of the HOMOs and LUMOs in the P dimer of C_6_-PyDI are shown in Fig. 3 ▸. We can see that the HOMOs and LUMOs are mainly localized on the pyrene core, and the introduction of alkyl chains has little influence on the frontier molecular orbital charge distributions. In contrast to the perfect face-to-face dimer, there exists an obvious displacement between two neighboring molecules of the P dimer along the molecular axis direction, which leads to a cancelation effect between the bonding and antibonding overlaps and a decrease in the effective coupling projected area. As shown in Fig. 3 ▸(*b*), we can see that the major contribution to the *V*_h_ value mainly comes from N⋯π interactions, and π⋯π interactions in the P dimer contribute little to the *V*_h_ value due to the cancellation effect. In comparison, the distribution character of the LUMO leads to intermolecular O⋯π interactions and partially effective π⋯π interactions in the P dimer, as show in Fig. 3 ▸(*d*), which explains the fact that the *V*_e_ value in the P dimer of C_6_-PyDI is about three times larger than the corresponding *V*_h_ value. Through the above analysis, we can conclude that the weak electronic couplings in the C_6_-PyDI crystal are mainly due to the large deviation of the P dimer from perfect face-to-face packing, which results in the compensation of bonding and antibonding interactions.

In the crystal structures of *t*-C_5_-PyDI and *t*-C_6_-PyDI, the molecules also pack into a lamellar motif; however, every two neighboring molecules form a molecular pair, and the distance between these molecular pairs is much longer than the intermolecular distance within the molecular pair. For example, the distance between *t*-C_6_-PyDI pairs is 11.629 Å, and the intermolecular distance in the *t*-C_6_-PyDI pair is only 3.519 Å. For the sake of discussion, the dimer with a small intermolecular distance, *i.e.* 3.519 Å, is defined as a P1 dimer; and a dimer with a larger distance, *i.e.* 11.629 Å, is defined as a P2 dimer. Our calculations show that the intermolecular electronic couplings in the P1 dimer are much larger than those in the P2 dimer, which is consistent with the intermolecular distances. As shown in Table 2 ▸, the *V*_h_ and *V*_e_ values in the P1 dimer of *t*-C_5_-PyDI are 91.1 and 152.3 meV, respectively, which are much stronger than those in the P2 dimer (1.5 and 0.2 meV); similarly, the *V*_h_ and *V*_e_ values in the P1 dimer of *t*-C_6_-PyDI are 90.5 and 145.7 meV, respectively; in comparison, both *V*_h_ and *V*_e_ values in the P2 dimer are less than 0.01 meV. In this case, the hole/electron transport mobility is primarily determined by the hopping rate between different P1 dimers. The weak electronic couplings in the P2 dimers of *t*-C_5_-PyDI and *t*-C_6_-PyDI explain their poor OFET properties.

### 3.3. Anisotropic mobility   

**Figure 4:**
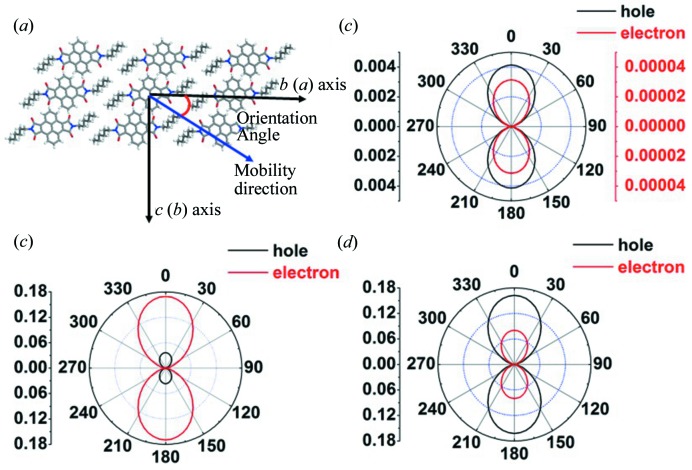
(*a*) Illustration of the orientation angle of the transistor channel relative to the crystallographic *b* axis for C_5_-PyDI and C_6_-PyDI and the orientation angle of the transistor channel relative to the crystallographic *a* axis for *t*-C_5_-PyDI. Calculated angle-resolved anisotropic hole (black line) and electron (red line) transport mobility as a function of orientation angle for (*b*) *t*-C_5_-PyDI, (*c*) C_6_-PyDI and (*d*) C_5_-PyDI.

The anisotropic hole-transfer and electron-transfer mobility values in the single crystals of C_6_-PyDI and C_5_-PyDI are shown in Fig. 4 ▸. It can be seen that their similar crystal structures result in the same angle dependence of mobility; both C_6_-PyDI and C_5_-PyDI show remarkable anisotropic behavior and the highest mobility values appear when the value of Φ is near 0/180°, that is, along the crystallographic *b* axis. This mobility distribution as a function of Φ is consistent with their one-dimensional molecular packing character. The ranges of mobility values of C_5_-PyDI and C_6_-PyDI estimated in the same layer are summarized in Table 3 ▸. We can see that the ranges of the hole- and electron-mobility values in C_5_-PyDI and C_6_-PyDI crystals agree well with the experimental measurements by Wu *et al.* (2017 ▸), which verifies the rationality of our computational method and strategy. Comparison of the predicted hole and electronic mobility values for C_5_-PyDI and C_6_-PyDI indicates that the holes in C_5_-PyDI are intrinsically more mobile than the holes in C_6_-PyDI; while for the electron, the mobility values in the crystal of C_5_-PyDI are obviously lower than those in C_6_-PyDI. Combined with the experimental results reported recently, it can be seen that the hole- and electron-transfer mobilities of C_5_-PyDI and C_6_-PyDI in the experiments of Wu *et al.* (2017 ▸) might have reached their optimum values as *n*-type or ambipolar materials.

For *t*-C_5_-PyDI, we simulated the angular resolution anisotropic mobility for both electron and hole transport. As shown in Fig. 4 ▸(*b*), it can be seen that the hole- and electron-transfer mobility values in the *ab* plane show similar anisotropic behavior: the highest and lowest mobility values were present at Φ = 0/180° and Φ = 90/270°, respectively. The maximum hole-transfer mobility value is only 0.004 cm^2^ s^−1^ V^−1^, and the maximum electron-transfer mobility value is less than 0.0001 cm^2^ s^−1^ V^−1^, which is consistent with the reported highest electron transport mobility (8.72×10^−5^ cm^2^ s^−1^ V^−1^). Although the introduction of the *t*-butyl group has very slight effects on the reorganization energy associated with charge-carrier transfer, the steric effect induced by its large volume dramatically changes the relative position between adjacent molecules, which has a large interference on the size of *V*_h_ and *V*_e_ values. As a result, the OFETs fabricated from *t*-C_5_-PyDI exhibited lower conductive performance.

### 3.4. Ionization potential and electronic affinity   

Aside from mobility, charge injection efficiency is also an important factor that affects the performance of an OFET device, especially for the ambipolar and *n*-channel OFETs. For OFETs, it requires that the electrode materials have work functions suited for the injection of holes/electrons into the HOMO/LUMO of semiconductor molecules. In molecular orbital theory approaches, the HOMO energy is related to the IP by Koopmanns’ theorem and the LUMO energy is used to estimate the electron affinity (−*E*_HOMO_ = IP and −*E*_LUMO_ = EA); however, the −*E*_HOMO_/−*E*_LUMO_ values are usually inconsistent with IP/EA values in the practical DFT calculations, partly due to the unknown ‘exact’ exchange-correlation functional. Previous calculations by Zhan *et al.* showed that the directly calculated vertical IPs are, on the whole, in good agreement with the corresponding experimental IPs (Zhan *et al.*, 2003 ▸). Thus, we select IP and EA values of the studied compounds as the evaluation parameters to analyze the charge-injection barrier of the OFET.

For the metal electrode, the key for efficient injection of charge-carrier is that the IP values should be close to or smaller than the work function of the metal electrode; and the EA values should be close to or larger than the work function of the metal electrode. Considering the thermal and oxidative stability of electron-transport materials, the suitable EA values need to be at least 2.8 eV (Newman *et al.*, 2004 ▸). The calculated IPs and EAs of C_5_-PyDI, C_6_-PyDI, *t*-C_5_-PyDI and *t*-C_6_-PyDI are shown in Table 4 ▸. We can see that the AIP values of C_5_-PyDI, C_6_-PyDI, *t*-C_5_-PyDI and *t*-C_6_-PyDI are larger than 7.0 eV. In this case, it needs a strong external electric field to overcome the high hole-injection barriers. It is noteworthy that the introduction of the *t*-butyl group in C_5_-PyDI and C_6_-PyDI could decrease their AIP values by 0.25 and 0.27 eV, respectively, which is favorable for hole injection. For the predicted EA values, it is observed that (i) all AEA values of C_5_-PyDI, C_6_-PyDI, *t*-C_5_-PyDI and *t*-C_6_-PyDI are larger than 1.9 eV, suggesting PyDI can be selected as electron-deficient frameworks for *n*-type or ambipolar semiconductors; and (ii) addition of the *t*-butyl group causes a slight decrease in AEA values of C_5_-PyDI and C_6_-PyDI, which is unfavorable to the electron-injection process.

### 3.5. Electronic spectra   

**Figure 5:**
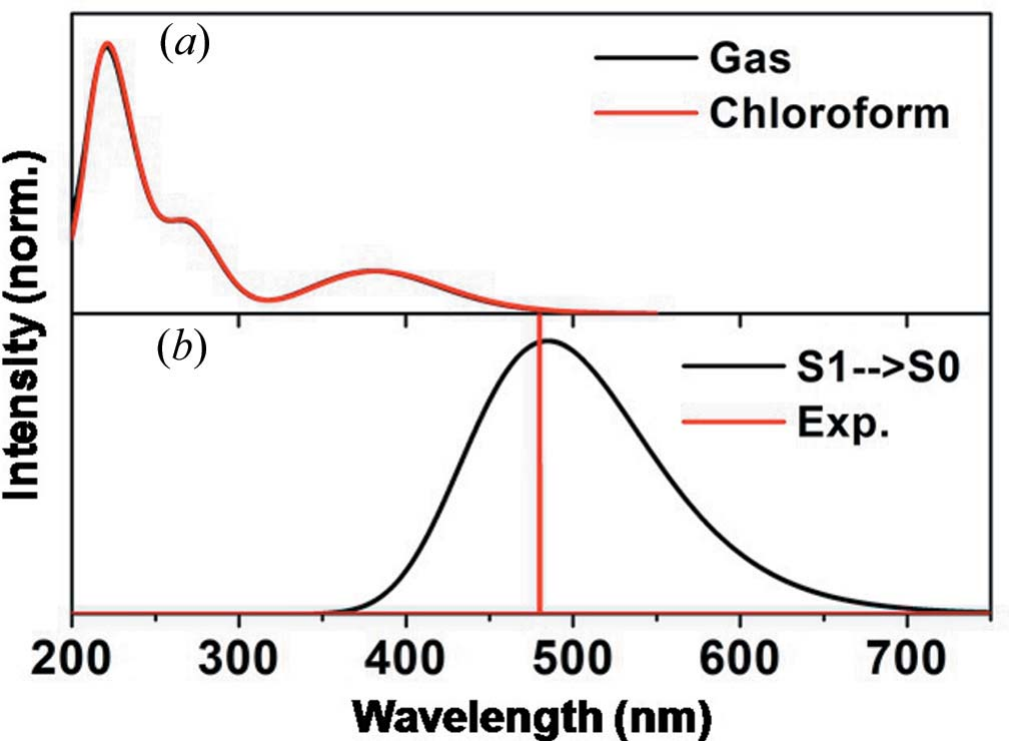
(*a*) Calculated absorption spectra of *t*-C_5_-PyDI in gas and in chloro­form. (*b*) Calculated fluorescence spectra of *t*-C_5_-PyDI in cyclo­hexane.

Based on the optimized geometries, we simulated the UV–vis absorption spectra of *t*-C_5_-PyDI in the gas phase and in chloro­form solvent, as shown in Fig. 5 ▸(*a*). It can be seen that the calculated absorption peak positions in chloro­form solvent are similar to the case in the gas phase. Their maximum absorption bands are observed in the near-UV region, which is consistent with the experimental excitation wavelength (270 nm) obtained by Wu *et al.* (2017 ▸). To gain a deeper understanding of the spectra character, the excited states and the frontier molecular orbitals (FMOs) were analyzed in detail, which can provide information about the nature of the excited-state conformations. For *t*-C_5_-PyDI in chloro­form, as shown in Table 5 ▸, there exists an *S*_0_→*S*_1_ transition at 392 nm, an *S*_0_→*S*_3_ transition at 350 nm and an intense *S*_0_→*S*_9_ transition at 271 nm. The *S*_1_ state of *t*-C_5_-PyDI is mainly formed by the transitions from the HOMO to the LUMO; the *S*_3_ and *S*_9_ states are predominantly formed by the transitions from HOMO-1 to the LUMO, and by the transitions from the HOMO to LUMO+2, respectively. Moreover, the fluorescence spectrum of *t*-C_5_-PyDI is also simulated and the results are shown in Fig. 5 ▸(*b*). It is found that the predicted fluorescence maximum of *t*-C_5_-PyDI, corresponding to the *S*_1_→*S*_0_ transition process, is located at 485 nm, which is quite close to the experimental value of 480 nm (Wu *et al.*, 2017 ▸).

## 4. Conclusions   

In this manuscript, we simulated anisotropic charge-transfer mobilities of C_5_-PyDI, C_6_-PyDI and *t*-C_5_-PyDI, and theoretically predicted the range of their mobility values, which provided reference for the performance optimization of an OFET based on these materials. We systematically analyzed the influences of the alkyl chain on the reorganization energies, crystal packing, electronic couplings and charge injection barrier of PyDI. It is found that the introduction of alkyl chain groups into PyDI has little effect on the reorganization energy, but increases the repulsive interactions of the backbone and thus affects the molecular packing in the crystal. As a result, the electrons in the *t*-C_5_-PyDI crystal are intrinsically less mobile than the electrons in the C_5_-PyDI crystal. Moreover, the alkyl chain substitution could decrease the electronic affinities and ionization potentials, which decreases the hole-injection barrier and improves the injection efficiency of the holes. We also simulated electronic spectra of *t*-C_5_-PyDI which reproduced the experimental absorption and fluorescence spectra well.

## Figures and Tables

**Table 1:** DFT-B3LYP/6-311G** calculated hole-transfer (λ_h_) and electron-transfer (λ_e_) reorganization energies of C_5_-PyDI, C_6_-PyDI, *t*-C_5_-PyDI and *t*-C_6_-PyDI by the APS approach

Molecular crystals	λ_h_ (eV)	λ_e_ (eV)
C_5_-PyDI	0.170	0.258
C6-PyDI	0.169	0.257
*t*-C_5_-PyDI	0.180	0.251
*t*-C_6_-PyDI	0.179	0.251

**Table 2:** Calculated electronic coupling terms *V*_h_ (hole transfer) and *V*_e_ (electron transfer) for the different hopping pathways in C_5_-PyDI, C_6_-PyDI, *t*-C_5_-PyDI and *t*-C_6_-PyDI crystals; *r* is the intermolecular center-to-center distance

Molecular crystals	Dimer types	*r* (Å)	*V*_h_ (meV)	*V*_e_ (meV)
C_5_-PyDI	P1 = P2	4.880	15.0	17.9
C_6_-PyDI	P1 = P2	4.795	7.2	26.3
*t*-C_5_-PyDI	P1	4.78	91.1	152.3
	P2	7.796	1.5	0.2
*t*-C_6_-PyDI	P1	3.519	90.5	145.7
	P2	11.629	0.01	0.01

**Table 3:** Theoretical hole-diffusion mobilities (μ_h_) and electron-diffusion mobilities (μ_e_) of C_5_-PyDI, C_6_-PyDI and *t*-C_5_-PyDI at room temperature (*T* = 300 K), and some experimental charge-transfer mobility values

	μ_h_ (theor.)	μ_e_ (theor.)	μ_e_ (exp.)†
C_5_-PyDI	0–0.16	0–0.080	0.19 ± 0.13
C_6_-PyDI	0–0.04	0–0.17	0.29 ± 0.12
*t*-C_5_-PyDI	0–0.004	(0–3) × 10^−5^	(8.72 ± 0.91)×10^−5^

† Wu *et al.* (2017 ▸).

**Table 4:** VIPs, AIPs, VEAs and AEAs calculated at the B3LYP/6-311** level (eV)

Molecules	VIP	AIP	VEA	AEA
C_5_-PyDI	7.59	7.51	1.87	2.00
C_6_-PyDI	7.59	7.51	1.88	2.01
*t*-C_5_-PyDI	7.34	7.25	1.84	1.96
*t*-C_6_-PyDI	7.32	7.23	1.83	1.95

**Table 5:** Electronic excitation energies (λ), oscillator strengths (*f*), corresponding compositions and the configuration interactions (CI) for *t*-C_5_-PyDI in gas and in cyclo­hexane

	Transition	λ(nm)	*f*	Composition	CI (%)
*t*-C_5_-PyDI (gas)	*S*_0_→*S*_1_	383	0.201	H→L	91
	*S*_0_→*S*_10_	268	0.471	H→L + 2	79
*t*-C_5_-PyDI (cyclo­hexane)	*S*_0_→*S*_1_	392	0.287	H→L	100
	*S*_0_→*S*_3_	350	0.129	H − 1→L	85
	*S*_0_→*S*_9_	271	0.595	H→L + 2	82
